# Development and Diagnostic Accuracy of a Novel Screening Tool for Early Detection of Pediatric Visual Impairment in Indonesian School-Aged Children

**DOI:** 10.3390/healthcare14091233

**Published:** 2026-05-03

**Authors:** Arya Ananda Indrajaya Lukmana, Tri Rahayu, Kianti Raisa Darusman, Ray Wagiu Basrowi, Nila Djuwita F. Moeloek

**Affiliations:** 1 Indonesia Health Development Center (IHDC), Jakarta 12950, Indonesia; aryalukmana08@gmail.com (A.A.I.L.); kiantiraisa@gmail.com (K.R.D.); nilafmoeloek@gmail.com (N.D.F.M.); 2 Indonesian Ophthalmologist Association, Perhimpunan Dokter Spesialis Mata Indonesia (PERDAMI), Jakarta 10320, Indonesia; 3 Department of Ophthalmology, Faculty of Medicine, Universitas Indonesia, Jakarta 10430, Indonesia; 4 JEC Eye Hospitals and Clinics, Jakarta 10310, Indonesia; 5 Laulima Society, Jakarta 12330, Indonesia; 6 Health Collaborative Center (HCC), Jakarta 11550, Indonesia; 7 Occupational Medicine Division, Department of Community Medicine, Faculty of Medicine, Universitas Indonesia, Jakarta 10430, Indonesia

**Keywords:** visual impairment, pediatric, refractive errors, screening tool, primary care, community ophthalmology, vision screening, visual impairment in children

## Abstract

**Highlights:**

**What are the main findings?**

- The CIPSEL Score (Cipete Selatan Score) is a newly developed 10-item questionnaire-based screening tool designed to detect visual impairment consistent with uncorrected refractive error in school-aged children, administrable by non-medical personnel in under-resourced primary care settings; in this study, involving 131 students from grades 3 to 5, estimated to be between 8 and 12 years of age based on the Indonesian standard school entry age system, the tool demonstrated strong diagnostic accuracy with an Area Under the Curve (AUC) of 0.887 (95% CI: 0.829–0.946) and a robust statistical association with clinical visual status (Cramér’s V = 0.534, *p* < 0.001).
- The instrument features an adaptable dual-cutoff system: a score of ≥3 maximizes sensitivity (96.2%) for population-level safety-first screening, while a score of ≥4 balances sensitivity (80.8%) and specificity (79.0%) for efficient clinical referral, enabling context-specific deployment across school and primary healthcare settings.

**What are the implications of the main findings?**

- The CIPSEL Score offers a scalable, low-cost, and culturally adapted screening solution that can be integrated into existing Indonesian school health programs and potentially replicated across other low- and middle-income countries (LMICs) where access to formal ophthalmic services remains limited.
- The proposed tiered risk stratification approach enables more nuanced clinical decision-making at the primary care level, allowing health providers to prioritize high-risk cases for immediate referral while establishing an active monitoring pathway for moderate-risk students, directly supporting WHO Vision 2030 targets for preventable visual impairment.

**Abstract:**

Background/Objectives: Uncorrected refractive errors (UREs) are a primary cause of preventable visual impairment in children globally, impacting education and quality of life. In Jakarta, prevalence has surged to 40% post-pandemic, categorizing it as a serious public health problem. This study aimed to develop and validate the CIPSEL questionnaire as a rapid, culturally adapted screening tool for identifying visual impairment consistent with possible UREs among Indonesian school-aged children. Methods: A cross-sectional study was conducted in South Jakarta with 131 students aged 8–12 years. The 10-item CIPSEL questionnaire, exploring visual behaviors and symptoms, was administered via face-to-face interviews. Visual acuity was assessed using a standard Snellen chart by medical personnel blinded to the questionnaire results. Diagnostic accuracy was assessed using receiver operating characteristic (ROC) curve analysis, with optimal thresholds determined via the Youden Index and the shortest distance to (0, 1). Results: Visual impairment was identified in 26 students (19.8%). Mean CIPSEL scores were significantly higher in students with visual impairment (4.73) compared to those with normal vision (1.95). ROC analysis showed considerable diagnostic accuracy with an AUC of 0.887 (95% CI: 0.829–0.946). A safety-first cutoff of 2.5 prioritized sensitivity (96.2%), while a balanced cutoff of 3.5 provided 80.8% sensitivity and 79.0% specificity. A tiered risk system (Low, Medium, and High) demonstrated a robust statistical association with actual clinical findings (Cramer’s V = 0.534, *p* < 0.001). Conclusions: CIPSEL is a reliable and scalable screening tool for the early detection of visual impairment in Indonesian children. Its tiered risk stratification framework facilitates nuanced clinical decision-making and efficient resource allocation in school-based settings. Its accessibility for non-medical personnel and potential for digital integration support national efforts toward universal eye health.

## 1. Introduction

Uncorrected refractive errors (UREs) remain the leading cause of preventable visual impairment across all age groups globally, with significant consequences in the paediatric population. Globally, an estimated two out of three individuals in low-income countries with distance vision impairment due to URE lack access to corrective eyeglasses, and the number of people requiring refractive error care is projected to increase substantially in the coming decade, with myopia alone expected to reach 3.36 billion cases by 2030, driven largely by modifiable lifestyle-related risk factors [1]. Uncorrected refractive errors can negatively impact educational opportunities, productivity, and the overall quality of life [2]. Myopia is the most common type of refractive error. Of the estimated US$244 billion in global productivity loss attributed to uncorrected myopia, Southeast Asia bears the heaviest proportional burden among all regions, underscoring the urgent need for scalable vision care interventions [3]. In the local context of Jakarta, the situation has become increasingly critical in the post-pandemic era. Recent studies indicate that the prevalence of refractive errors among elementary school children has surged to 40%, a sharp increase from pre-pandemic estimates, which ranged between 22% and 28%. This surge is largely attributed to lifestyle shifts during COVID-19 social restrictions, characterized by excessive gadget use, prolonged online learning, and a significant lack of outdoor activities. With prevalence rates now exceeding the 30% threshold, pediatric refractive error in Jakarta is categorized as a serious public health problem [4].

Learning begins in childhood, making accurate vision crucial for a child’s learning ability. Vision is vital in shaping an individual’s future, and career planning for youth often relies on visual acuity, especially in fields such as the Navy, the military, railways, and aviation [5]. The negative effects of URE can be more pronounced in children, as poor visual experiences from URE can lead to irreversible amblyopia, which not only affects vision but also results in social, educational, and economic challenges in adulthood. Impaired vision, along with reduced stereoacuity and contrast sensitivity, hampers job performance and capabilities. This decline in quality of life has a detrimental effect on psychosocial well-being. Thus, focusing on screening and early detection initiatives within the community is essential [6].

Early disease detection in primary care settings, such as Puskesmas (Public Health Center), faces various challenges including limited staff, time, patient awareness, and resource constraints. Time constraints and competing health priorities also hinder primary care providers’ ability to conduct comprehensive screenings [7]. Gaps in parental awareness contribute to delays in addressing vision issues. Research conducted in Saudi Arabia revealed that 60% of parents did not bring their children for regular eye check-ups, and 70% were not informed about amblyopia [8]. A study in Granada, Spain, found that 56.5% of parents were unaware of the significant consequences of high myopia on ocular health [9].

Early screening enables detection of children’s visual problems, allowing timely intervention to prevent long-term impairment. In schools, correcting refractive errors through eyeglasses improves academic performance, demonstrating the importance of early vision care [10].

In a Focus Group Discussion conducted by Basrowi et al. [11] elementary teachers from a public school in Jakarta highlighted the importance of implementing school-based refractive error screening programs. They agreed that such programs could effectively identify refractive errors in students. The teachers supported standardized screening tests with accessible guidelines for non-health professionals, believing this would aid the learning process [11].

To address this gap, there is a critical need for a rapid, parent-friendly, accessible, and resource-efficient screening tool. The CIPSEL questionnaire was created to fulfil this need, serving as a symptom-based screening tool to identify children at risk for refractive errors. It was adapted from the validated instrument proposed by Vasudevan et al. in their study “Development and validation of a screening tool for the identification of refractive errors among school-going children in Tamil Nadu, India.” [12]. This study aims to develop and validate the CIPSEL questionnaire as a screening tool for pediatric visual impairment consistent with UREs by evaluating its diagnostic performance through AUC-ROC analysis, determining an optimal cutoff score, and assessing the sensitivity, specificity, and reliability of this scoring system using advanced statistical methods.

## 2. Materials and Methods

This study employed an observational analytic design using a cross-sectional approach to evaluate the diagnostic accuracy of a newly developed screening tool—the CIPSEL questionnaire—for detecting refractive errors among school-aged children. The study was conducted on 9–10 October 2024, at an elementary school in South Cipete, South Jakarta. Participants were students aged 8–12 years, specifically from grades 3 to 5, who were present and assented verbally to participate during the school health screening session. The age range of 8–12 years was estimated based on the standard Indonesian primary school entry age (7 years) and the grade levels of participating students (grades 3–5), consistent with the Ministry of Education’s national schooling guidelines. Individual age data were not recorded in accordance with the anonymization protocol. Sample size was calculated using the Buderer formula for diagnostic accuracy studies, based on an expected sensitivity of 84% derived from the reference instrument by Vasudevan et al., a disease prevalence of 40%, a margin of error of 10%, and a 95% confidence level (z = 1.96), yielding a minimum required sample of 130 students. The 40% prevalence figure was selected as a conservative worst-case estimate to ensure adequate sample size for diagnostic accuracy analysis. The actual observed prevalence of 19.8% in this single-school pilot study reflects the specific characteristics of the study setting and should not be interpreted as a population-level prevalence estimate. The final sample of 131 students met this requirement. Exclusion criteria included students who declined to participate or were absent on the day of data collection. The CIPSEL questionnaire was developed by adapting a validated instrument by Vasudevan et al. [12], modified for cultural relevance and language suitability. The adaptation process involved a strategic selection of the most significant and predictive items from the original study. These items were then translated into Indonesian to maintain linguistic clarity. By focusing on high-impact visual behaviors and symptoms, such as squinting and headaches, the CIPSEL tool was refined into a 10-item format optimized for ease of use by non-medical personnel in local settings. These 10 yes/no items explore visual behaviour and refractive error-related symptoms, including squinting, headaches, and visual copying habits. The complete 10-item CIPSEL questionnaire, including the scoring direction for each item, is presented in Table 1. Each item is scored dichotomously (Yes = 1, No = 0), except for Item 5, for which a “No” response is scored as 1, reflecting its inverse relationship with refractive error risk. The total CIPSEL score ranges from 0 to 10. Item 9 (‘If you have worn glasses before, do you wear them throughout the day?’) was administered to all 131 participants. Students who had never worn glasses responded ‘No’ to this item, a semantically valid response reflecting the absence of corrective lens use, and were accordingly assigned a score of 0. No items were omitted or skipped, and no proportional score adjustment was applied. All total CIPSEL scores were therefore calculated as the sum of all 10 items (range: 0–10), applied uniformly across the entire sample.

During the screening, the questionnaire was administered via face-to-face interviews with students by trained healthcare workers. Visual acuity was assessed using a standard Snellen chart positioned at 6 m by trained medical personnel (doctors or nurses) from the local Puskesmas.

To minimize potential bias, the personnel conducting the visual acuity tests were blinded to the CIPSEL questionnaire results, and vice versa. Visual acuity worse than 6/9 in the better-seeing eye, without correction, was classified as having a refractive error, while normal vision was coded as ‘0’. Data were collected using anonymized Google Forms, cleaned in Microsoft Excel, and analysed using IBM SPSS Statistics for Macintosh, Version 25.0 (IBM Corp., Armonk, NY, USA). Descriptive statistics summarized population characteristics, and receiver operating characteristic (ROC) curve analysis was performed to assess diagnostic accuracy. The optimal cut-off score was determined using the maximum Youden Index and Nearest Distance to (0, 1) and risk stratification was conducted based on total CIPSEL scores. Additional analyses included Chi-square tests, Cramer’s V for association strength, and internal consistency via Cronbach’s alpha.

This study was conducted as part of a routine school health screening program implemented by Cipete Selatan Primary Healthcare Centre (Puskesmas Pembantu Kelurahan Cipete Selatan) in accordance with Jakarta Provincial Regulation No. 34 Year 2024 and Circular Letter No. 63/SE/2024 issued by the Jakarta Health Office. Ethical review and approval were waived in accordance with the 2021 National Health Research Ethics Standards and Guidelines issued by the Indonesian National Committee for Health Research Ethics (Komite Etik Penelitian dan Pengembangan Kesehatan Nasional, KEPPKN), specifically under criteria outlined in Chapter III, Section B, Point 2a of the KEPPKN 2021 Guidelines: (1) Exempt criterion (iii), as the study was conducted within a normal educational setting involving standard health screening practices; (2) Exempt criterion (iv), as data were collected via survey and interview procedures without recording individually identifiable information; and (3) the public program evaluation criterion (research conducted under specific government authority to evaluate a public health service program, with no significant physical invasion or privacy interference) [13]. Demographic data such as age and gender were not individually recorded, as the screening activity followed a public health protocol that prioritized anonymity and program efficiency. All participants were primary school students aged approximately 8–12 years from grades 3 to 5, and the absence of demographic identifiers aligns with the ethical principles of anonymity and minimal data collection in routine school health screenings.

## 3. Results

### 3.1. Participant Characteristics

A total of 131 students from grades 3 to 5 participated in the study as part of a routine school health screening program (Table 2). These students were estimated to be between 8 and 12 years old based on their grade level. Among all participants, 26 students (19.8%) were classified as having abnormal vision based on unaided visual acuity worse than 6/9 in the better-seeing eye.

### 3.2. CIPSEL Score Distribution

The CIPSEL scores among participants ranged from 0 to 9 points, with a mean score of 2.5 (SD = 1.947), a median of 2.0, and a mode of 1.0 points, which was the most frequently occurring score (*n* = 31, 23.66%). When stratified by visual status, notable differences in score distributions were observed between the two groups, as presented in Table 3. Students with normal vision recorded a mean CIPSEL score of 1.95 (SD = 1.614, median = 2.00, range 0–7), while students with visual impairment recorded a substantially higher mean score of 4.73 (SD = 1.564, median = 4.50, range 2–9), suggesting a clear separation between the two groups in terms of symptom burden.

**Figure 1:**
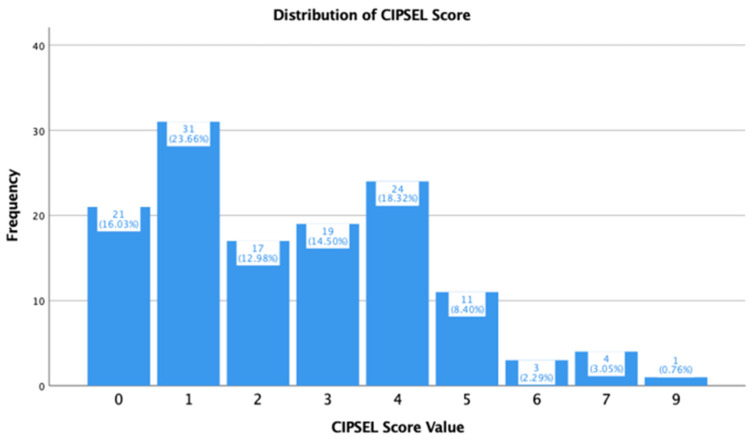
Frequency Distribution of CIPSEL Scores.

**Figure 2:**
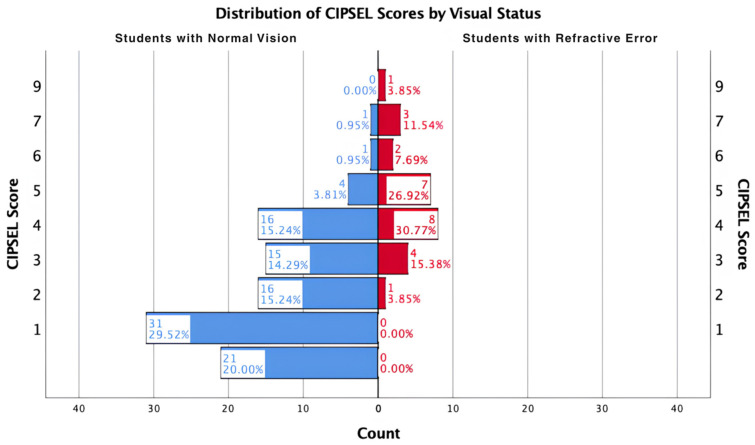
Distribution of CIPSEL Scores by Visual Status.

A bar chart illustrates the frequency distribution of CIPSEL scores across all participants (Figure 1), while Figure 2 shows the distribution of scores stratified by visual status.

### 3.3. Diagnostic Performance of the CIPSEL Questionnaire (ROC Curve Analysis and Cut-Off Determination)

**Figure 3:**
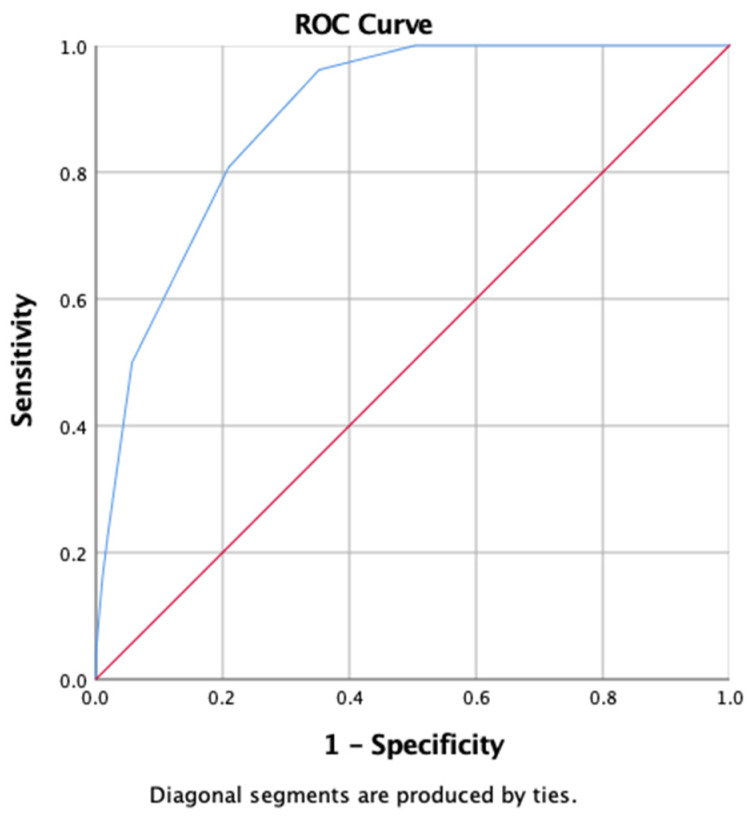
ROC Curve of the CIPSEL Questionnaire. The blue line represents the diagnostic performance of the questionnaire, while the red diagonal line represents the line of no discrimination (reference line, AUC = 0.5).

The diagnostic accuracy of the CIPSEL questionnaire was assessed using a receiver operating characteristic (ROC) curve. The area under the curve (AUC) was 0.887, with a 95% confidence interval of 0.829 to 0.946 (Figure 3).

Cut-off analysis identified two potential optimal thresholds for the CIPSEL score based on different statistical criteria. A cut-off score of 2.5 was identified as the optimal threshold according to the Youden Index (0.610), yielding a high sensitivity of 96.2% and a specificity of 64.8%. Alternatively, a cut-off score of 3.5 was determined to be the optimal threshold based on the shortest distance to the point (0, 1) in the ROC space (0.285), providing a more balanced diagnostic performance with a sensitivity of 80.8% and a specificity of 79.0%. An overview of the performance metrics across various cut-off points is presented in Table 4.

### 3.4. Instrument Reliability and Association with Visual Status

Based on the ROC curve analysis, a tiered risk stratification system was developed to categorize students into three distinct risk levels. This classification utilizes the identified optimal cut-offs to maximize screening safety while maintaining referral efficiency. The Low-Risk category, comprising scores of 0–2, is anchored by the Youden Index cut-off of 2.5, offering a high sensitivity of 96.2% to ensure that children with a minimal risk of visual impairment are accurately identified. The Medium-Risk category, defined by a score of 3, represents a monitoring zone between the two optimal statistical thresholds where clinical suspicion remains moderate. High Risk, encompassing scores of 4–10, aligns with the Shortest Distance to the point (0, 1) criteria of 3.5 and above, where specificity increases significantly to over 79.0%, indicating a high probability of actual visual impairment. A Pearson Chi-square test was conducted to examine the association between the three CIPSEL risk categories and the participants’ actual visual status, as detailed in Table 5. The analysis revealed a statistically significant association (Pearson Chi-Square = 37.415, *p* < 0.001), with a strong linear-by-linear association showing that the probability of visual impairment increases proportionally with the CIPSEL risk level. Table 5 presents the distribution of students within the Low (0–2), Medium (3), and High (4–10) risk categories across their respective normal vision and refractive error status. The strength of this association, measured using Cramer’s V, was 0.534, indicating a robust relationship between the tiered questionnaire scores and clinical findings.

## 4. Discussion

This study evaluated the diagnostic performance of the CIPSEL questionnaire, a culturally adapted screening tool for detecting reduced visual acuity consistent with UREs among school-aged children in an urban Indonesian setting. A total of 131 students from grades 3 to 5 (approximately aged 8–12 years) participated in the study. Among them, 26 students (19.8%) were classified as having abnormal visual acuity (worse than 6/9 in the better-seeing eye). This figure reflects a considerable burden of visual impairment, and is consistent with prior global estimates, which indicate that approximately over 20% of primary school-aged children worldwide are affected by myopia, with higher prevalence reported in East and Southeast Asia [14]. The prevalence observed in this study may also be partly attributed to the study’s urban setting, a private school located in South Jakarta, where children are more likely to experience increased screen exposure and near-work activities, along with limited outdoor time, all of which are well-established risk factors for the development and progression of myopia [15,16]. The decision to use 20/30 as the threshold for visual impairment aligns with the American Academy of Ophthalmology (AAO) “Critical Line” evaluation. For children aged 60 months and older, the AAO specifies 20/30 as the age-dependent line they are expected to pass normally. Utilizing this “critical line” is more effective than threshold testing 20/20, as younger children may fail the stricter line due to limited attention rather than actual vision deficits. This approach provides a practical balance for mass screening: it reliably identifies significant concerns while preventing the healthcare system from being overwhelmed by over-referrals [17]. It is important to note that visual acuity measurement via the Snellen chart, while standard for mass screening, does not constitute a definitive diagnosis of URE in the absence of cycloplegic refraction. Therefore, findings in this study reflect visual impairment consistent with possible URE rather than confirmed refractive error. It is essential to emphasize that a positive CIPSEL screen does not constitute a diagnosis. The tool is intended as a preliminary triage layer, identifying children who warrant referral for formal clinical evaluation, including best-corrected visual acuity testing and, where indicated, cycloplegic refraction, rather than as a replacement for ophthalmological assessment. A fundamental methodological consideration concerns the use of uncorrected Snellen visual acuity as the reference standard, rather than cycloplegic refraction, which is widely regarded as the gold standard for diagnosing refractive errors in children [18]. This limitation was acknowledged a priori, and the study outcome variable was consequently framed as ‘visual impairment consistent with possible URE’ rather than ‘confirmed refractive error.’ It is recognized that non-cycloplegic Snellen testing may underestimate accommodative hyperopia, as children with significant hyperopia may compensate through active accommodation and achieve normal visual acuity despite clinically relevant refractive error, and may conversely overestimate visual impairment in children with accommodative excess or spasm, where transiently reduced distance acuity reflects a functional rather than structural deficit, or in those with limited cooperation, attention, or comprehension of the test task during mass screening [19,20,21]. However, this pragmatic choice reflects real-world primary care constraints in Indonesia, where cycloplegic refraction is not feasible in mass school-based screening. Importantly, this same limitation is shared by virtually all large-scale school vision screening programs globally, including those endorsed by the WHO, which rely on visual acuity as the primary screening metric because of its practicality [22]. The AUC of 0.887, therefore, reflects CIPSEL’s performance against the same reference standard used operationally in the target setting, a programmatically valid benchmark even if not the biological gold standard.

The CIPSEL instrument demonstrated considerable diagnostic accuracy, with an area under the curve (AUC) of 0.887 (95% CI: 0.829–0.946). According to established classification criteria [23], AUC values ≥ 0.9 reflect excellent discriminatory capability, while values between 0.8 and 0.9 are considered considerable. The optimal cut-off point was determined using two commonly applied methods: the Youden Index and the distance to the (0, 1) point on the ROC curve. The Youden Index (sensitivity + specificity − 1) identifies the cut-off with the highest combined diagnostic performance; thus, the highest Youden value indicates the most balanced threshold. Meanwhile, the distance to (0, 1) represents the geometric closeness of each cut-off to the ideal point of perfect sensitivity and specificity (100%, 100%) on the ROC plot—the smallest distance value suggests the best-performing cut-off [24]. In this study, these two methods yielded slightly different optimal thresholds, reflecting the classic trade-off between sensitivity and specificity in diagnostic testing. The Youden Index identified a score of 2.5 as the optimal threshold (J = 0.610), prioritizing a high sensitivity of 96.2%. Conversely, the shortest distance to (0, 1) suggested a score of 3.5 (d = 0.285), which offered a more balanced diagnostic performance with a sensitivity of 80.8% and a specificity of 79.0%. To bridge the gap between these two statistically valid points, a tiered risk stratification system was adopted to reconcile the different priorities of each method into a clinically applicable framework. The Low-Risk category, comprising scores of 0–2, anchors its lower bound at the Youden Index-derived threshold of 2.5, ensuring a “safety-first” screening approach that maximizes the detection rate and ensures nearly all children with potential refractive errors are flagged for further evaluation. Scores of 3 represent a Medium Risk or “divergence zone” between the two statistical methods; identifying this as a moderate-risk category provides a practical monitoring buffer, allowing for scheduled re-screenings. For children in the Medium-Risk category (score = 3), a structured monitoring approach is recommended rather than immediate referral or discharge. A re-screening interval of 3–6 months is proposed, allowing sufficient time to observe whether symptoms stabilize or progress, while avoiding unnecessary burden on specialist services. Suppose symptoms worsen before the scheduled re-screening, such as increased squinting, declining academic performance, or new visual complaints, prompt referral to an ophthalmologist is warranted. The High-Risk category, encompassing scores of 4–10, aligns with the shortest distance to the (0, 1) point criteria (threshold > 3.5) and emphasizes specificity. At this level, the likelihood of a true visual impairment status is significantly higher, justifying prompt referral to eye care specialists. This tiered system is particularly advantageous for large-scale, school-based screenings in urban settings where healthcare resources must be managed efficiently, as it transforms the binary “pass/fail” result into a more nuanced clinical decision tool. The robustness of this classification is further supported by a Cramer’s V of 0.534 (*p* < 0.001), which confirms a strong association between these risk categories and actual clinical outcomes.

Translating these robust metrics into a practical framework, this tiered risk stratification facilitates nuanced clinical decision-making within the school environment. By categorizing children into low, moderate, and high-risk groups, the system enables flexible resource allocation, ensuring that interventions are prioritized for those with the highest clinical suspicion while providing a safety net for those in the lower-risk brackets. Each risk level is linked to specific follow-up recommendations derived from the tool’s diagnostic performance at each respective threshold, as detailed in Table 6.

Internal consistency, measured by Cronbach’s alpha = 0.649, was modest yet acceptable given the tool’s binary format and early-stage development. According to Nunnally’s psychometric standards, values above 0.6 can be considered adequate in exploratory instruments [25].

Compared with the original instrument by Vasudevan et al. [12], CIPSEL retains core predictive elements while simplifying the format to 10 contextually relevant items. While the original tool reported a sensitivity of 84% and a specificity of 63%, CIPSEL offers superior performance through its tiered thresholds. At the 2.5 cut-off, CIPSEL achieves a significantly higher sensitivity of 96.2% and a comparable specificity of 64.8%, making it a safer option for mass screening. In contrast, the 3.5 cut-off, derived from the shortest distance to the (0, 1) point, optimizes specificity at 79.0%. This more balanced threshold is better suited for targeted referrals, helping to avoid unnecessary clinical workload by prioritizing students with a higher probability of visual impairment. This trade-off, guided by statistically optimal thresholds, reflects a rational approach to screening policy-making where priorities can shift between maximum sensitivity and referral efficiency based on available follow-up resources [26]. Additionally, unlike Snellen chart testing or autorefractors, which require equipment and trained personnel, CIPSEL relies on observed visual behaviours and self-reported symptoms—making it more accessible and scalable for mass screening in low-resource school environments [27]. CIPSEL’s design philosophy and clinical utility become further apparent when contrasted with other validated pediatric eye assessment instruments in the literature. The Pediatric Eye Questionnaires (PedEyeQ), developed by Hatt et al., represent a comprehensive, psychometrically rigorous instrument assessing eye-related quality of life (ER-QOL) and functional vision across multiple unidimensional domains, including functional vision, social impact, frustration/worry, and parental concern, across three separate age cohorts (0–4, 5–11, and 12–17 years), with distinct proxy and child-reported versions totaling up to 42 items per version [28]. While the PedEyeQ offers exceptional depth for clinical and research settings, its multi-domain, multi-version architecture renders it impractical for rapid, large-scale school-based screening by non-medical personnel. In contrast, CIPSEL’s 10-item binary format was deliberately streamlined to prioritize implementation feasibility over psychometric comprehensiveness, making it more suitable as a first-line triage tool in resource-limited environments. The Convergence Insufficiency Symptom Survey (CISS), another well-established pediatric visual symptom questionnaire, demonstrates excellent diagnostic accuracy for convergence insufficiency (CI) in children aged 9–18 years, with a sensitivity of 96% and specificity of 88% at a cutoff of ≥16, and an intraclass correlation of 0.77, indicating good test–retest reliability [29]. However, the CISS was specifically designed and validated for CI, a binocular vision disorder, rather than for refractive error or uncorrected visual acuity deficits. Its 15-item Likert-scale format also introduces greater respondent burden compared to CIPSEL’s binary yes/no structure, which is particularly advantageous when screening younger children or when administered by teachers without clinical training. CIPSEL therefore fills a distinct gap as a purpose-built, symptom-based instrument targeting reduced visual acuity consistent with possible URE, rather than binocular dysfunction. A further point of comparison is offered by a web-based visual acuity and refractive error self-assessment tool evaluated by Claessens et al. in myopic children aged 6 years and older. This home-based digital platform, performed with parental assistance, demonstrated good agreement with clinic-based Snellen chart testing and a sensitivity of 94% and specificity of 71% for detecting VA poorer than 0.10 logMAR, metrics comparable to CIPSEL’s performance at the 2.5 cutoff [30]. However, the web-based refractive error algorithm overestimated myopia progression and required recalibration for the pediatric population, highlighting the ongoing technical limitations of self-administered digital tools in this age group. Such platforms presuppose adequate digital literacy, device availability, and home internet access, prerequisites that cannot be assumed across Indonesia’s diverse socioeconomic landscape. CIPSEL, by contrast, requires no digital infrastructure at the point of screening and can be administered verbally in any school setting, making it more equitable and immediately deployable within Indonesia’s national school health program framework.

The implications for community ophthalmology are significant. CIPSEL aligns with the WHO’s call for scalable, low-cost screening tools integrated into primary healthcare systems [31,32]. Its simplicity allows potential use by non-specialist personnel—teachers, community health workers, or even parents—thereby increasing coverage in areas underserved by ophthalmologists. This is consistent with findings from a Focus Group Discussion conducted by Basrowi et al. (2024) involving elementary school teachers in Jakarta, who emphasized the need for simple, school-based screening instruments to aid early detection efforts [11]. Furthermore, as technology access continues to expand, CIPSEL also has the potential for digital adaptation or self-administered formats. A preliminary concept for a mobile-based application version of the tool is included in the Supplementary Materials to further explore this direction.

The current study was conducted among children aged 8–12 years (grades 3–5), a population in whom the critical period for amblyopia development has largely concluded. While CIPSEL demonstrates strong screening performance in this age group, the greatest opportunity for preventing permanent vision loss lies in earlier detection, ideally before the age of 7–8 years, when neuroplasticity of the visual cortex allows for effective amblyopia treatment [33]. Future development should therefore prioritize the adaptation of CIPSEL for younger cohorts, particularly children aged 4–6 years. A version tailored for this age group would require significant modification: the questionnaire format would need to shift from self-report to a proxy-report instrument completed by parents or caregivers, and items would need to be redesigned around observable behaviors accessible to non-clinical observers, given the limited verbal and cognitive capacity of preschool-aged children.

Nevertheless, several limitations must be acknowledged to provide a balanced interpretation. First, the study was conducted in a single school with a modest sample size (*n* = 131), potentially limiting generalizability. The absence of individually recorded sex and age data, while consistent with the anonymization protocol of the government-mandated screening program, precludes sex-stratified or age-stratified subgroup analyses. Given that the prevalence and symptomatology of refractive errors may differ between sexes and across specific age cohorts within the 8–12-year range, future studies should incorporate these variables under appropriate data protection frameworks to enable more granular analysis. As a single-center pilot study conducted in one urban private school in South Jakarta, generalizability to rural, public schools, or lower socioeconomic settings remains to be established. The study was designed as an initial validation study, not a population survey, and the sample size was calculated for diagnostic accuracy studies specifically, not for prevalence estimation. Second, the lack of cycloplegic refraction, considered the gold standard in pediatric diagnosis [18], may have led to misclassification of certain visual conditions. Consequently, the outcome variable in this study represents reduced visual acuity as a proxy for URE, and a confirmed refractive error status should be established through subsequent clinical examination. Third, demographic data such as sex and exact age were not individually recorded due to anonymization protocols, restricting subgroup analysis. While the internal consistency was acceptable, it indicates room for item refinement and psychometric enhancement in future iterations. Another consideration is that the current cultural adaptation of the CIPSEL instrument was strategically streamlined to prioritize immediate clinical utility and practical relevance within the Indonesian school context. While the tool has demonstrated robust diagnostic performance, future iterations could be further enhanced by adhering to an even more extensive, multi-phase adaptation and translation protocol. Ideally, such a process would encompass formal forward-translation and its subsequent synthesis, independent back-translation, committee-led harmonization, and rigorous pre-testing, concluding with a comprehensive analysis of its field-tested psychometric properties [34].

Furthermore, although the tool was developed with professional clinical oversight, involving a broader multidisciplinary team of both content specialists and linguistic experts in future phases could offer even deeper conceptual and idiomatic equivalence.

Despite these limitations, the study offers a compelling case for CIPSEL as a cost-effective, scalable, and locally relevant tool for early detection of refractive errors in Indonesian schoolchildren. Its integration into existing school health workflows and potential digital transformation can contribute significantly to national efforts to reduce childhood visual impairment, especially in line with WHO’s 2030 goals for universal eye health [35]. Moreover, early identification may help mitigate the estimated $244 billion global productivity loss associated with uncorrected myopia, highlighting both the health and economic value of such tools [3].

Beyond its current application in urban primary schools, the CIPSEL questionnaire holds significant potential for deployment in diverse and resource-constrained settings. Its reliance on observable visual behaviours and self-reported symptoms, rather than specialized ophthalmic equipment, makes it uniquely suited for rural or remote areas where access to ophthalmologists and diagnostic tools like Snellen charts is limited. Furthermore, the tool’s simplicity enables its adoption by a wide range of non-medical stakeholders, including parents for home-based preliminary checks and community health workers within the broader primary healthcare (Puskesmas) network. The flexibility of the dual-cutoff system also allows health administrators to tailor the tool for high-volume triage in public health campaigns or as a preliminary screening layer in digital telehealth platforms. Future initiatives should prioritize validating CIPSEL across various geographic and socioeconomic landscapes to establish its universal utility as a first-line defense against pediatric visual impairment.

Future research should focus on external validation across diverse populations and geographic settings to ensure the generalizability of the CIPSEL instrument within Indonesia’s heterogeneous demographics. To further elevate the tool’s psychometric robustness, subsequent studies should implement a rigorous, multi-phase cultural adaptation protocol, including forward and back-translation, synthesis, harmonization, and cognitive pre-testing, ideally conducted by a multidisciplinary team of ophthalmologists, public health experts, and linguistic specialists. Such a comprehensive approach will ensure maximum semantic and conceptual equivalence for various local dialects and cultural contexts. Additionally, future investigations should aim for the refinement of individual questionnaire items to further improve internal consistency and diagnostic precision. The incorporation of gold-standard diagnostic methods, such as cycloplegic refraction, in subsequent validation studies will be essential to provide a more definitive comparison for clinical accuracy. There is also an opportunity to explore the longitudinal tracking of students in the “Medium Risk” or “Transition Zone” (Score 3) to determine the most effective re-screening intervals and long-term outcomes for this specific group.

Real-world testing in integrated school-based and primary care workflows will further clarify CIPSEL’s practical utility in resource-limited environments. Supported by the strong initial association findings in this study, a refined tool like CIPSEL holds promise not only as an academic innovation but also as a scalable, community-centered solution to the persistent burden of uncorrected refractive errors in children.

## 5. Conclusions

The CIPSEL questionnaire demonstrated considerable diagnostic accuracy and a robust statistical association with actual visual status, providing a practical and linguistically adapted solution for the early detection of visual impairment among Indonesian primary school children. With an AUC of 0.887 (95% CI: 0.829–0.946), the tool offers a versatile screening framework through its dual-cutoff strategy. An optimal threshold of 2.5 provides a high sensitivity of 96.2%, making it an ideal “safety-first” filter for broad screening. A threshold of 3.5 yields a more balanced performance with a sensitivity of 80.8% and specificity of 79.0%, which is better suited for targeted referrals to optimize clinical resources. A significant contribution of this study is the development of a tiered risk stratification system, categorizing students into Low-Risk (0–2), Medium-Risk (3), and High-Risk (≥4) groups. This classification showed a strong relationship with clinical findings (Cramer’s V = 0.534, *p* < 0.001), transforming binary results into a nuanced clinical decision tool. The tool’s reliance on observed visual behaviors and self-reported symptoms ensures its accessibility to non-medical personnel, such as teachers and community health workers, thereby enhancing its reach in resource-limited or urban settings. The potential for digital adaptation further reinforces CIPSEL’s relevance for large-scale school health initiatives.

While the current version provides a solid foundation, continued refinement through a more exhaustive multi-phase cultural adaptation protocol, external validation across diverse geographic settings, and comparison with gold-standard methods like cycloplegic refraction are essential for future research. Ultimately, CIPSEL holds strong potential as a scalable, community-centered instrument to support both national and global efforts toward universal eye health and the reduction in uncorrected refractive errors in children.

## 6. Patents

While no patents have been granted at the time of this publication, the proprietary diagnostic workflow, risk stratification algorithm, and digital implementation framework of the CIPSEL tool described in this study are currently in the developmental phase for patent application filing. The authors are formalizing intellectual property protection to ensure the standardized and ethical deployment of this novel screening methodology within primary healthcare and school-based settings.

## Figures and Tables

**Table 1:** The CIPSEL Questionnaire: Items, Original Indonesian Phrasing, and Scoring Direction.

No.	Item (English Translation)	Original Item (Bahasa Indonesia)	Response Scored as 1 (Risk-Positive)
1	Do you prefer to sit in the front row because you cannot see the board clearly?	Apakah kamu lebih suka duduk di barisan depan karena tidak bisa melihat papan tulis dengan jelas?	Yes
2	Do you often write words incorrectly when copying from the board?	Apakah kamu sering salah menulis kata-kata saat menyalin dari papan tulis?	Yes
3	Do you prefer to copy notes from a friend rather than writing directly from the board?	Apakah kamu lebih suka menyalin catatan dari teman daripada menulis langsung dari papan tulis?	Yes
4	Do you frequently have headaches after school, more than 3 times a week?	Apakah kamu sering sakit kepala sepulang sekolah, lebih dari 3 kali dalam seminggu?	Yes
5	Do you play outdoors every day for more than one hour?	Apakah kamu bermain di luar rumah setiap hari selama lebih dari satu jam?	No
6	Do you like watching TV at a very close distance (less than 30 cm)?	Apakah kamu suka menonton TV dengan jarak yang sangat dekat (kurang dari 30 cm)?	Yes
7	Do you often squint your eyes to see things clearly?	Apakah kamu sering menyipitkan mata untuk melihat sesuatu dengan jelas?	Yes
8	Have you ever worn glasses before?	Apakah kamu pernah memakai kacamata sebelumnya?	Yes
9	If you have worn glasses before, do you wear them throughout the day?	Jika pernah pakai kacamata, apakah kamu memakainya sepanjang hari?	Yes
10	Has anyone ever told you that your eyes appear crossed or misaligned?	Apakah ada orang yang pernah bilang kalau matamu juling atau tidak lurus?	Yes

Note: Each item is scored dichotomously: the Risk-Positive response (column 4) is awarded 1 point; the alternative response is awarded 0 points. Item 5 is an inverse item, where “No” reflects a risk-positive behaviour (insufficient outdoor activity). Total scores range from 0–10. The original items were developed in Bahasa Indonesia to ensure linguistic clarity and cultural appropriateness for the target population. Item 9 was administered to all participants. Students who had never worn glasses (Item 8 = No) responded ‘No’ to Item 9 and received a score of 0 for this item, consistent with the universal 10-item scoring structure (range: 0–10).

**Table 2:** Summary of visual acuity outcomes among participating students.

Variable	*n*	%
Total participants	131	100%
Normal vision ^1^	105	80.2%
Visual impairment ^2^	26	19.8%

^1^ Visual Acuity ≥ (better than) 6/9 in better seeing eye. ^2^ Visual Acuity < (worse than) 6/9 in better seeing eye.

**Table 3:** Descriptive Analysis of CIPSEL Scores by Visual Status.

Visual Status		CIPSEL Score Descriptive Statistics
Student with Normal Vision	Mean	1.95
	Median	2
	Std. Deviation	1.614
	Minimum	0
	Maximum	7
Student with Visual Impairment	Mean	4.73
	Median	4.5
	Std. Deviation	1.564
	Minimum	2
	Maximum	9

**Table 4:** Diagnostic Accuracy of CIPSEL Scores at Different Cut-off Scores.

Cut Off	Sensitivity	Specificity	Youden Index	Distances to (0, 1)
−1	100.00%	0.00%	0	1
0.5	100.00%	20.00%	0.2	0.8
1.5	100.00%	49.50%	0.495	0.505
2.5	96.20%	64.80%	0.610 *	0.354
3.5	80.80%	79.00%	0.598	0.285 **
4.5	50.00%	94.30%	0.443	0.503
5.5	23.10%	98.10%	0.212	0.77
6.5	15.40%	99.00%	0.144	0.846
8	3.80%	100.00%	0.038	0.962
10	0.00%	100.00%	0	1

* Indicates the optimal cut-off determined by the highest Youden Index (J = 0.610). ** Indicates the optimal cut-off, determined by the shortest distance to the point (0, 1) (d = 0.285).

**Table 5:** Statistical Association Between Tiered CIPSEL Risk Categories and Visual Status (*n* = 131).

CIPSEL Risk Category	Students with Normal Vision (*n* = 105)	Students with Visual Impairment (*n* = 26)	Pearson Chi Square (*df*)	*p*-Value	Cramer’s V
Low Risk (Score 0–2)	68 (98.6%)	1 (1.4%)	37.415 (2)	<0.001	0.534
Medium Risk (Score 3)	15 (78.9%)	4 (21.1%)			
High Risk (Score 4–10)	22 (51.2%)	21 (48.8%)			

Note: Percentages represent the proportion within each CIPSEL Risk Category (Row %).

**Table 6:** CIPSEL Risk Stratification and Recommended Clinical Actions.

CIPSEL Score	Risk Category	Sensitivity	Specificity	Suggested Action
0–2	Low Risk	96.2%	64.8%	No action needed; routine checks only.
3	Medium Risk	Transition Zone		Re-screen within 3–6 months; refer immediately if symptoms worsen before scheduled re-screening.
≥4	High Risk	80.8%	79.0%	Refer to ophthalmologist.

## Data Availability

The data presented in this study are available in the Supplementary Material of this article, accessible at https://doi.org/10.17605/OSF.IO/4NVB5. The Supplementary Materials include the CIPSEL questionnaire and screening form, survey results, raw and processed data previews, SPSS output, supporting regulatory documents, and event documentation. Further inquiries can be directed to the corresponding authors.

## Supplementary Materials

The following supporting information can be downloaded at https://doi.org/10.17605/OSF.IO/4NVB5, accessed on 30 April 2026. File S1: CIPSEL Score Questionnaire and Screening Form; File S2: Survey and Form Results; File S3: Preview of CIPSEL Score Raw Data; File S4: CIPSEL Score Processed Data and SPSS Output; File S5: Jakarta Provincial Regulation No. 34 Year 2024 and Circular Letter No. 63/SE/2024 issued by the Jakarta Provincial Health Office (Dinas Kesehatan Provinsi DKI Jakarta); File S6: School Health Screening Event and Team Photograph & Documentation (Figure S1); Link S1: Official CIPSEL Score Website (https://s.id/CipselSkor, accessed on 30 April 2026); Link S2: Application Concept Prototype (https://s.id/appcipsel, accessed on 30 April 2026).

## Abbreviations

The following abbreviations are used in this manuscript:

URE	Uncorrected Refractive Error
CIPSEL	Cerah Indra Penglihatan Anak Selamat
AUC	Area Under the Curve
ROC	Receiver Operating Characteristic
VA	Visual Acuity
CI	Confidence Interval/Convergence Insufficiency
WHO	World Health Organization
AAO	American Academy of Ophthalmology
KEPPKN	Komite Etik Penelitian dan Pengembangan Kesehatan Nasional
CISS	Convergence Insufficiency Symptom Survey
PedEyeQ	Pediatric Eye Questionnaire
